# Next-generation single-cycle respiratory syncytial virus vaccines with increased type I interferon induction yield robust systemic and mucosal responses in mice

**DOI:** 10.3389/fimmu.2026.1778423

**Published:** 2026-04-10

**Authors:** Patil Basavaraju Nanjegowdu, Jeeviya Murugesan, Megolhubino Terhüja, Pramila Lamichhane, Valerie McElliott, Antonius G. P. Oomens

**Affiliations:** 1 Department of Veterinary Pathobiology, College of Veterinary Medicine, Oklahoma State University, Stillwater, OK, United States; 2 Neovaxsyn, Inc., Ames, IA, United States; 3 RNA Viruses Section, Laboratory of Infectious Diseases, National Institute of Allergy and Infectious Diseases, National Institutes of Health, Bethesda, MD, United States

**Keywords:** interferon, matrix protein, Mnull, NS1 protein, respiratory syncytial virus, RSV, vaccine

## Abstract

We previously demonstrated that vaccination with RSV-Mnull, a prototype single-cycle live vaccine lacking the matrix (M) gene, generated anti-viral serum IgG and memory T cell responses, and reduced challenge virus shedding and pulmonary dysfunction in mice. Here we further characterized the response to RSV-Mnull, and designed and tested second generation Mnull vaccines. In mice, prime-boost vaccination with RSV-Mnull generated pre-fusion (preF) and attachment protein (G) -specific serum IgG and lung IgA, and protected from lung pathology, showing that a single-cycle live vaccine was effective in this model. In an effort to enhance efficacy for future human application, second generation Mnull vaccines were designed, in which nonstructural protein 1 (NS1), a known interferon (IFN) antagonist, was relocated to reduce expression. In addition, the G or F genes were moved to the first genome position (RSV-Mnull/G1 and RSV-Mnull/F1 respectively). *In vitro*, RSV-Mnull/G1 and RSV-Mnull/F1 showed reduced NS1 levels and increased IFN-β induction, whereas IFN-λ levels were not affected. Viruses with relocated NS1 also displayed enhanced anti-viral state in uninfected cells. RSV-Mnull/G1 induced higher levels of anti-G serum antibodies (Abs), whereas RSV-Mnull/F1 increased the ratio of anti-preF:anti-G IgG Abs. RSV-Mnull induced lower levels of lung IgA than wildtype RSV; However, relocation of NS1 increased early IgA induction and restored IgA levels to those seen with wildtype RSV. These findings suggest differences in G and F presentation or processing and Ab induction, and indicate that the genomic location of NS1, G, and F can impact/improve IgG and IgA levels and timing. All vaccines induced Abs that neutralized RSV *in vitro* and protected animals against a high challenge dose of wildtype RSV. In terms of protection against lung pathology, we did not see major improvements of RSV-Mnull/G1 and RSV-Mnull/F1 over the RSV-Mnull prototype, potentially due to limitations of the model. Nevertheless, RSV-Mnull/G1 and RSV-Mnull/F1 elicited high Ab levels against both major antigens, enhanced serum anti-G IgG or lung anti-F IgA levels, and protected mice from lung pathology in spite of single-cycle replication. Thus, the single-cycle approach has potential to be a platform for development of safe and efficacious live RSV vaccines.

## Introduction

Respiratory syncytial virus (RSV) is the single-largest viral cause of bronchiolitis in infants and children, and results in >100,000 deaths/year in children worldwide (1–3). More than a decade ago, an RSV vaccine was designated a WHO priority (4). In the past few years, the FDA approved fusion (F) protein based vaccines for the elderly (Arexvy, Abrysvo, mRESVIA) and for pregnant women (Abrysvo) to protect newborns via passively acquired antibodies (Abs). In addition, new Abs with extended half-lives have been approved (Beyfortus, Enflonzia) for use in infants and children at risk for RSV infection. However, a safe and effective vaccine for the pediatric population is not available, and RSV remains a major worldwide medical burden for infants and children (1, 4–10). Live intranasal (IN) vaccines have long been considered an attractive approach because: a) live vaccines induce a broadly protective multi-antigenic response, avoiding dependence on a singular antigen for protection and the associated potential for immune escape; b) when applied intranasally, live vaccines elicit both systemic and mucosal responses - the latter will help protect from replication in the upper respiratory tract (11, 12) and dissemination of natural virus in the population; and c) IN vaccines may be less sensitive to inactivation by maternal IgG Abs than intramuscular vaccines (7, 13, 14), possibly due to poor transudation of IgG to the upper respiratory tract (15, 16). In addition, in the case of RSV, live vaccines are also the only pediatric vaccines that do not prime for vaccine-enhanced disease (VED) after challenge with RSV (7, 17), a phenomenon encountered in the 1960s with formalin-inactivated vaccine. Live RSV also has unique challenges, as the virus expresses several virulence factors that interfere with the host response. Live vaccines will likely need modifications to temper this host antagonism in order to induce effective and long-lasting immune responses. For young infants, immunologic immaturity and associated immune biases present further challenges (18). Finally, trials of live-attenuated RSV vaccines in children have so far failed to achieve a proper balance of efficacy and safety (14, 19–21), including a recent vaccine (SP0125; aimed at children aged between 6 and 22 months) which was dropped due to anticipated insufficient efficacy (22).

We previously generated live single-cycle RSV vaccines (23–25). These vaccines were not specifically targeted to one age group but may overcome some known challenges associated with vaccination of RSV-naive individuals. The single-cycle approach endows a live vaccine with a reliable and stable safety component, i.e. a block in cell-cell transmission, which prevents virus transmission in a vaccinated individual and potential reversal to a more virulent virus. At the same time, live single-cycle vaccines are not attenuated for genomic replication and thus *de novo* synthesize high levels of antigens in infected cells. For RSV-Mnull, its single-cycle phenotype is based on the absence of the matrix (M) protein gene from the viral genome (25). The latter study demonstrated that a single vaccination with RSV-Mnull generated anti-viral serum IgG and memory T cell responses, and reduced challenge virus shedding and pulmonary dysfunction in mice. Here we further characterized the response induced by RSV-Mnull, and designed 2nd generation Mnull vaccines in an effort to enhance efficacy. For the latter we modified expression of a well-characterized host antagonist, the non-structural protein1 (NS1). NS1 is believed to be the main player in interferon (IFN) antagonism, although non-structural protein2 (NS2), M and the attachment protein (G) have all been shown to impact the IFN response (26–30). Outright deletion on NS1 was reported to block generation of high-titer RSV stocks (31, 32), and hence we reduced NS1 expression levels by genome relocation to the eighth position. The rationale for targeting NS1 and improved IFN induction was based on literature that shows that in animals infected with RSV (and other viruses), low or delayed type I IFN as a consequence of immune immaturity, can lead to unbalanced immune responses that may have contributed to VED, such as T helper 2 (Th2) bias, poor CD4 T cell and dendritic cell (DC) maturation, and weak development of the adaptive response and memory T cells (30, 33–47). In contrast, an increase in early levels of type I IFN was shown to improve the outcome of RSV disease in mice and humans (18, 48–51).

In addition to relocating NS1, we moved the G or F gene to the first genome position with the intent to enhance expression levels and thereby potentially vaccine efficacy. Relocating G to the first genome position was previously shown to enhance anti-G serum IgG levels (23). These modified Mnull viruses, along with the previously published prototype RSV-Mnull as a comparison, were investigated *in vitro* and in mice for their vaccine potential. In the process, we also further characterized the RSV-Mnull prototype itself. The results of our study suggest that a live single-cycle approach may serve as a platform for the development of safe and protective RSV vaccines that engage mucosal immunity in both the upper and lower respiratory tracts. Mucosal immune responses at these sites is an established correlate of protection for respiratory viruses, including RSV, influenza virus and SARS-CoV-2, where local Ab and innate immune responses contribute to reduced viral replication and disease severity (11, 47, 52, 53). In addition, this platform can be modified to induce higher levels of type I IFN, which may further enhance antiviral immunity and improve vaccine efficacy.

## Materials and methods

### Cells and Abs

Vero 76 (Vero), HEp-2 and A549 cells were acquired from American Type Culture Collection and grown at 37 °C in advanced DMEM supplemented with 4% fetal bovine serum, 50 units/ml penicillin, 50 μg/ml streptomycin, and 2 mM glutamax. HEp-2-based cells expressing RSV M protein (H2-M) were previously generated (25) and were maintained in the same medium plus 0.2 mg/ml of hygromycin every other passage. D25 and Mota were kindly provided by Jason McLellan, (University of Texas at Austin, Austin, TX). MAb L9 (54) was provided by Ed Walsh (University of Rochester School of Medicine, Rochester, NY). Mab B023 (Anti-N Ab) was purchased from Bio-Rad. For western blot, a rabbit polyclonal anti-NS1 peptide serum was kindly provided by Michael Teng (University of South Florida, Tampa, FL). Anti–glyceraldehyde-3-phosphate dehydrogenase (GAPDH) Ab was purchased from Invitrogen. IgG- and IgA-specific horseradish peroxidase (HRP)-conjugated secondary Abs were purchased from Southern Biotech.

### Construction of viral cDNAs, recovery of infectious virus, and production of virus stocks

cDNAs were based on the RSV A2 strain and were generated using standard molecular biology methods as previously described (24, 55, 56), using remote-cutting enzyme BsmBI for exact replacement of open reading frames (ORF). For surrogate wildtype virus RSV-rWT, the SH ORF was replaced by that of EGFP (56). For Mnull prototype virus RSV-Mnull, the SH ORF was substituted with that of EGFP and the M ORF replaced with a Tet transactivator (tTA) sequence (25, 56). From the RSV-Mnull cDNA, the NS1 ORF was moved to the eighth genome position to generate Mnull viruses with reduced NS1 expression. Additionally, we moved either the F or G ORF to the first genome position to enhance expression. RSV virus expressing horseradish peroxidase (HRP) (RSV-HRP), in which the SH ORF was replaced with that of HRP, was previously reported (24). Viruses were recovered from cDNAs as described previously (24, 25). Briefly, BHK-21 cells expressing T7 polymerase were transfected with engineered cDNAs and support plasmids containing an internal ribosome entry site and expressing nucleoprotein N, phosphoprotein P, transcription elongation factor M2-1, and polymerase L. For RSV-Mnull viruses, a plasmid expressing M protein was also included to compensate for the missing M gene. After 70 hours of incubation at 33 °C, supernatants from transfected cells were transferred to H2-M helper cells and incubated for another 4–5 days at 37 °C. Low passage seed stocks were generated by scraping cells, removing cell debris by low-speed centrifugation, and freezing at -80 °C. All virus stocks used in experiments were generated from these low-passage seed stocks. RSV-rWT virus was generated in HEp-2 cells whereas Mnull viruses were produced in H2-M helper cells. Vaccine batches were generated by infecting cells with seed stock at low MOI and incubating at 37 °C for 4–5 days. From infected cells, virus stocks were prepared by scraping the cells, vigorous pipetting, and low-speed centrifugation to remove cell debris. Supernatants were then pelleted by ultra centrifugation at 10,000 x g for one hour through a 25% sucrose cushion, washed, and re-suspended in OPTIMEM containing 5% sucrose and 100 mM MgSO4. Tubes were then flash-frozen in liquid nitrogen, and stored at -80 °C. For challenge studies, batches of the recombinant fully wildtype A2 virus were produced similarly, with the challenge virus being generated in Vero cells to minimize responses to host proteins. Virus stocks were titrated in duplicate using plaque assays, with plaques scored based on GFP expression observed from days 3 to 4. The changes made in the viral genomes were confirmed by bulk sequencing the modified genome areas after RNA purification from infected cells and RT-PCR.

### Cell ELISA to measure F and G levels on the surface of infected cells

The surface expression of major antigens F and G was assessed as previously described (23). In brief, HEp-2 cells were infected at 1 PFU/cell, and protein expression was examined at 26 hours post infection (hpi) by cell ELISA. Primary Abs specific to RSV, including L9 (anti-G), D25 (anti-preF only), and Mota (anti-F), were incubated on unfixed cells (to maintain native conformation) for 2 hours at RT. N protein levels were used as an infection control. After washing away unbound Abs, cells were fixed with freshly dissolved 4% paraformaldehyde for 5 minutes at room temperature. 0.1% BSA was included as a blocking agent in both Ab incubation steps. HRP levels were measured by adding O-phenylenediamine dihydrochloride (OPD) based ELISA substrate. The reaction was stopped by adding 3M sulfuric acid, and the optical density at 490 nm (OD490) was measured using a Versamax plate reader (Molecular Devices, CA, USA).

### Cell-cell transmission to demonstrate single-cycle phenotype

To verify deficiency in cell-cell transmission in regular cell types, HEp-2 and H2-M cells were plated side-by-side and infected at 0.01 PFU/cell. Following infection, GFP expression was monitored daily from day 1–5 pi, as a measure of virus transmission from cell to cell. Cells were fixed with freshly-dissolved 4% paraformaldehyde for 5 min, stained with Hoechst, and photographed on a Nikon TE2000 inverted fluorescent microscope at 100x magnification.

### Quantitative reverse-transcription PCR

A549 cells were infected at 1 PFU/cell. Total RNA was harvested from cells at 24 hpi using the EZNA Total RNA Kit I (Omega Bio-tek Inc., GA, USA). The purity of total RNA from the samples was evaluated and quantified using the NanoDrop ND-1000 spectrophotometer. For individual reactions, 1 μg of RNA was purified from genomic DNA with RNase-free DNase and subjected to cDNA synthesis with Oligo dT_18_ and random hexamer primers using Protoscript II reverse transcriptase (New England Biolabs Inc., MA, USA) according to the manufacturer’s instructions. qRT-PCR was then performed in duplicates using the PowerUp SYBR Green PCR Master Mix (Thermo Fisher Scientific, DE, USA). In a 96 well plate, each reaction mixture (20 μl) consisted of 10 μl SYBR green PCR master mix, 0.5 µL of 10 µM for each forward and reverse primer of the targets of interest (See **Table 1**; designed with primer blast), 5 μl of the cDNA template and 4 μl nuclease-free water. For negative control samples, RNase/DNase-free H_2_O was added instead of the cDNA template. PCR reactions were performed and analyzed using the QuantStudio 6 Pro (Thermo Fisher Scientific, DE, USA) Real-Time PCR System. The thermal cycling conditions were as follows: initial denaturation at 50 °C for 2 min, 95 °C for 5 min, followed by 40 cycles of amplification at 95°C for 15s, 60°C for 60s, followed by melt curve at 95 °C for 15s, 60°C for 60s, and 95 °C for 1s. Melting curve analysis of every qPCR reaction was conducted after each cycle. The fold change was calculated using ΔΔCt quantification method by measuring the target gene expression in the samples relative to the positive control (RSV-rWT); Results were then normalized to infection control RSV N levels. All data are shown as mean ± SD. Significance was determined by one way ANOVA.

**Table 1:** Primer pairs used for quantitative real-time PCR (qRT-PCR).

Gene	Forward primer (5’ - 3’)	Reverse primer (5’ - 3’)
hGAPDH	GAAGGTGAAGGTCGGAGTCAAC	CATGGGTGGAATCATATTGGAA
hIFN β1	AGTGTCAGAAGCTCCTGTGG	CATAGATGGTCAATGCGGCG
hIFN λ2/3	CCTGAATTGTGTTGCCAGCG	TTTCCTGGAGGTGAGTTGGATT
RSV N	CAGAATACAGGCATGACTCTCC	CGGCTGTAAGACCAGATCTG
RSV NS1	ATGGCATTGTGTTTGTGCATGT	GACCATTAGGTTGAGAGCAATGTG

### Western blot analysis

A549 cells were infected at 1 PFU/cell and incubated for 24 hours. Cells were lysed with Laemmli buffer, scraped and boiled for 5 min. Equal volumes (12 µL) of lysates were electrophoresed using reducing 10% SDS-PAGE, followed by transfer to a PVDF membrane (Immobilon, MilliporeSigma, MA, USA) using a semidry Bio-Rad apparatus, and blocked with 5% non-fat milk in TBS-Tween. The membranes were incubated for 2 hours with primary Ab (anti-NS1 and anti-GAPDH) and 1 hour with secondary Abs. Following each incubation, membranes were washed with TBS-Tween three times for 5 minutes. HRP was detected using Clarity Western ECL Substrate (Bio-Rad Laboratories, CA, USA) on an Amersham Imager 600 (GE). GAPDH was included as a loading control. The experiment was carried out twice with similar results.

### Measurement of IFNs in supernatants

To measure the production of IFN-β and IFN-λ3, A549 cells were infected at 1 PFU/cell and incubated at 37 °C. Supernatants were collected at 24 hpi, centrifugated at 21,130 x g for 10 min to clear debris, and stored at −80 °C. The levels of IFN-β and IFN-λ3 in the supernatants were determined using the human IFN-β and human IFN-λ3 DuoSet ELISA kits (R&D Systems, MN, USA), following manufacturer’s instructions. In brief, plates were coated with capture Abs and incubated overnight at RT. The coated plates were then washed and blocked with 5% non-fat milk. Standards and diluted samples were added to the plates in triplicate and incubated for 2 hours. After washing, biotinylated detection Abs were added and incubated for 1 hour, followed by washing and 30 minutes of incubation with a Streptavidin-HRP solution. The HRP signal was developed and read as described above for cell ELISA. IFN-β and IFN-λ3 concentrations were calculated based on the standard curves using a 4-parameter logistic function fitted to 8 data points from the standards.

### Anti-viral state assay

To evaluate the impact of altered IFN responses on viral replication, we developed an RSV-HRP inhibition bioassay based on Vlaspolder 1989 and Voigt 2013 (57, 58). A549 cells were infected at 1 PFU/cell and incubated at 37 °C. At 24 hpi, supernatants were harvested and centrifugated at 21,130 x g for 10 min to clear debris. Cleared supernatants were transferred to a 96-well plate containing freshly plated A549 cells (2x 10^4^ cells/well) and incubated at 37 °C for another 24 hours. Supernatants were then removed and cells were infected with RSV-HRP at 1 PFU/cell. At 24 hours post-infection, HRP expression was measured as described above, as an indicator of anti-viral state. As controls for the assay, a mock sample was included, which received supernatants from uninfected cells, as well as a mock-mock sample, which received supernatants from uninfected cells and was not infected with RSV-HRP.

### Ethics statement for use of vertebrate animals

Female BALB/c mice were purchased from Jackson Laboratory (Bar Harbor, ME, USA). All mouse studies were approved by the Institutional Animal Care and Use Committee at Oklahoma State University (OSU Stillwater animal assurance number A3722-01). Experiments were conducted according to the guidelines of the Office of Laboratory Animal Welfare and the Public Health Service Policy on Humane Care and Use of Laboratory Animals. Animals were euthanized as per guidelines of the American Veterinary Medical Association. For intranasal immunization, animals were anesthetized using isoflurane delivered in oxygen. Anesthesia was induced in an induction chamber with an oxygen flow rate of 2 L/min and isoflurane set to 5%. Following attainment of a surgical plane of anesthesia, isoflurane was reduced to 2–3% for maintenance, with oxygen flow adjusted to 0.8 L/min. Animals were vaccinated promptly under anesthesia and were continuously monitored until full recovery. For euthanasia, mice were deeply anesthetized by intraperitoneal administration of ketamine (200 mg/kg) and xylazine (10 mg/kg), followed by cervical dislocation in accordance with American Veterinary Medical Association guidelines.

### Animal study design and procedures

Eight-week-old female BALB/c mice (n=5 per group) were anesthetized by isoflurane inhalation and intranasally vaccinated with a prime and boost dose of 0.5 million PFU per dose, administered three weeks apart. This regimen was established following **Supplementary Figure S1**, in which the most optimal dose was determined for production of anti-viral Abs. Blood and perfused lung samples were collected 21 days post-boost to measure IgG in serum and IgA Ab levels in the lungs and for *in vitro* neutralization analysis. Lungs were homogenized in PBS containing protease inhibitors using a Precellys bead homogenizer (Bertin technologies, France) (2×20s at 8500 rpm), and the suspension was clarified by centrifugation. For the challenge study, mice were vaccinated as before and intranasally challenged with 2 million PFU of RSV-rWT five weeks post-boost. Mice were euthanized five days after the challenge, and lungs were collected for histopathology. Lungs were fixed in 10% neutral buffered formalin, processed through graded alcohols and xylene, paraffin-embedded, sectioned at 4 µm, and stained with hematoxylin and eosin (H&E) (Sigma, St. Louis, MO, USA). The slides were scored by board-certified (ACVP) veterinary pathologists blinded to study groups, examining RSV-induced pathology parameters on a scale from 0 (no lesions) to 3 (marked lesions). Assessed parameters included interstitial pneumonia (alveolar septa thickness, leukocytes in the alveolar space), edema (flooding of lymph vessels around blood vessels and airways), mucus, and neutrophil influx. For each group (n = 5), average total pathology scores were determined by adding up all the parameter scores from individual mice and dividing by five.

### Determination of RSV-specific IgG and IgA in serum and lungs by ELISA (pre-F and G)

RSV specific IgG and IgA Abs in mouse sera and lung samples were measured by ELISA as previously described (23, 24, 59). Briefly, 96 well plates were incubated overnight with purified G or preF proteins. Coated plates were washed and blocked with 5% non-fat milk. For IgG Ab in mouse serum, samples were serially diluted in 3-fold steps, with a starting dilution of 1:100. For IgA Ab levels in lungs, lung homogenates were two-fold serially diluted starting from undiluted. Samples were incubated for 2 hours at RT. This was followed by incubation with a HRP conjugated goat-anti-mouse secondary Ab (either IgG or IgA specific) and washing steps. HRP levels were measured as described in the cell ELISA section. Ab curves were analyzed using GraphPad Prism to determine the Area Under the Curve (AUC). Mean AUCs were then compared using one-way ANOVA. The reported values represent the mean and standard errors from five samples for each group.

### *In vitro* neutralization assay

A recombinant virus that expresses HRP (RSV-HRP) was previously generated and used for neutralization assays (24). Neutralizing Ab titers were measured using HEp-2 cells as a function of HRP gene expression after infection with RSV-HRP. Serum samples from each mouse group (n = 5) were pooled and heat-inactivated at 56 °C for 30 minutes. No exogenous complement was added, ensuring the neutralization is specific to complement-independent Ab neutralization. RSV-HRP (300 PFU per sample) was incubated with serum samples serially diluted threefold from 1:10 to 1:21,870 dilutions. After a 1-hour incubation, the mixtures were transferred to freshly plated HEp-2 cells, incubated for 1 hour, and then replaced with fresh media. After 48 hours of incubation at 37°C, HRP expression was measured as described above. The dilutions that resulted in 50% neutralization were determined using a non-linear curve fit (GraphPad Prism). Sera from mock vaccinated mice and random mice IgGs were used as negative controls.

### Bio-plex cytokine assay

BALB/c mice were immunized as above. Four days post-boost, lungs were harvested and homogenized as described above. Cytokine protein levels in each sample were assessed using 50 μL of homogenized lung tissue with the Bio-Plex Pro Mouse Cytokine Th1/Th2 immunoassay kit (Bio-Rad Laboratories, CA, USA) following the manufacturer’s instructions, on a Plex^®^ 200 multiplex detection system (Bio-Rad). Samples were tested in duplicate, and data were acquired using Bio-Plex Manager Software (Bio-Rad). The cytokines examined included GM-CSF, IFN-γ, IL-2, IL-4, IL-5, IL-10, IL-12, and TNF-α.

### Statistical analyses

Statistical analyses were performed using GraphPad Prism software. Data are presented as the mean ± SD unless otherwise stated. Comparisons between multiple groups were performed using one-way ANOVA followed by Tukey’s multiple comparisons test to compare each group mean with every other group mean. Statistical significance is indicated as *p < 0.05, **p < 0.01, ***p < 0.001, and ****p < 0.0001. All *in vitro* experiments were performed with n = 3 biological replicates and were carried out at least twice with similar results. All *in vivo* experiments were performed with n = 5 animals per group.

## Results

### Construction and characterization of modified Mnull viruses

**Figure 1:**
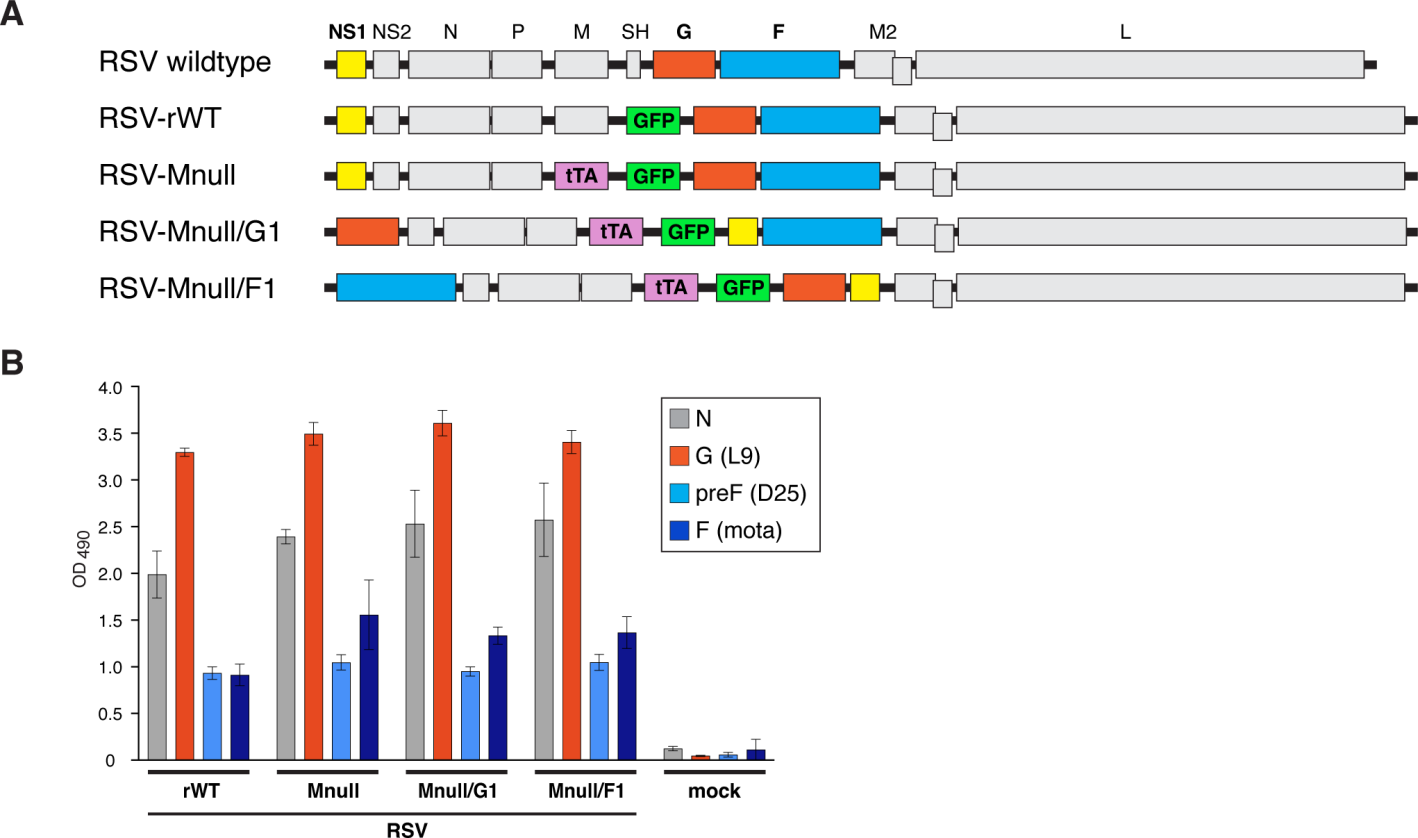
Composition of viral genomes and surface expression of viral glycoproteins. **(A)** Schematic of viral genomes. The genome of wildtype RSV is shown on top (not to scale). RSV-rWT, in which the SH ORF is replaced with that of GFP, serves as surrogate wildtype virus. In RSV-Mnull, the M ORF is replaced with that of Tet transactivator (tTA) gene (expression of tTA drives expression of M in the production cell line). Two new Mnull vaccines were generated by moving NS1 to the eighth position. In one case, the G ORF was moved to the first genome position (RSV-Mnull/G1). In the second experimental virus, the F ORF was moved to the first genome position (RSV-Mnull/F1). **(B)** Expression of viral glycoproteins on the surface of infected cells. HEp-2 cells were infected with various Mnull viruses, or left uninfected (mock), and protein expression was examined at 26 hpi by cell ELISA. Primary Abs specific to G (L9), preF (D25), and total F (Mota) were incubated on infected cells prior to fixation. Cells were then washed, fixed, and incubated with an HRP-conjugated secondary Ab. To detect N (infection control), cells were fixed and permeabilized prior to incubation with anti-N Ab. Error bars are the standard deviation of the mean of triplicate samples.

A previously engineered surrogate wild-type virus, RSV-rWT (**Figure 1A**), elicited a robust short-term immune response and protected mice from RSV pathology without triggering VED (24), and served as a positive control in this study. RSV-Mnull, our single-cycle vaccine prototype, induced Ab and T cell responses on par with RSV-rWT and prevented shedding of challenge virus from the upper respiratory tract (25). RSV-Mnull was however primarily designed to provide a stable safety phenotype, and we continued to develop the vaccine in anticipation of optimum efficacy needed in humans. In an effort to improve the prototype’s efficacy, we focused on enhancing type I IFN induction, and specifically IFN-β. This is based on literature mentioned in the Introduction that highlights the importance of IFNs in shaping the innate and adaptive immune responses and the ability of increased early levels of type I IFN to improve the outcome of RSV disease in mice and humans. To achieve this, we generated two new RSV-Mnull vaccines with reduced NS1 activity by relocating NS1 to the eighth genome position, based on the polar gradient of gene transcription observed in RSV (60, 61). The rationale for relocating NS1 was based on previous findings in which full deletion of NS1 negatively impacted the ability to generate high titer virus, presumably through IFN-independent mechanisms (31, 32). Relocating NS1 to a downstream genome position also allowed moving either the F or G ORF to the first genome position to enhance expression. In the case of G, relocation was shown to enhance anti-G Ab levels after vaccination (23). The composition of the experimental RSV-Mnull viruses is shown in **Figure 1A**. The virus with NS1 in 8th position and F in the first position was designated RSV-Mnull/F1; the one with NS1 in 8th position and G in the first position was designated RSV-Mnull/G1. We also mutated G residue methionine 48 in all viruses to leucine, to ablate the expression of secreted G, which can serve as a virulence factor (50, 62). These viruses were generated from cDNA by reverse genetics, and the modifications were confirmed by RT-PCR and bulk sequencing.

The surface expression of the major viral antigens F and G in infected HEp-2 cells was examined at 26 hpi by cell ELISA. To detect the G protein, Ab L9 was used. The primary Abs used for F were D25 (anti-preF) or Mota (anti-F). N protein levels (Ab B023) were determined to serve as an infection control to verify approximately equal infection rates. All Abs, except N, were incubated on unfixed cells, to ensure the native conformation of preF protein. Results showed that all viruses exhibited similar levels of F, G and N proteins, indicating that viruses replicated at similar rates and that the genome modifications did not adversely affect the expression of major viral antigens (**Figure 1B**).

### Mnull viruses are blocked in cell-cell transmission

**Figure 2:**
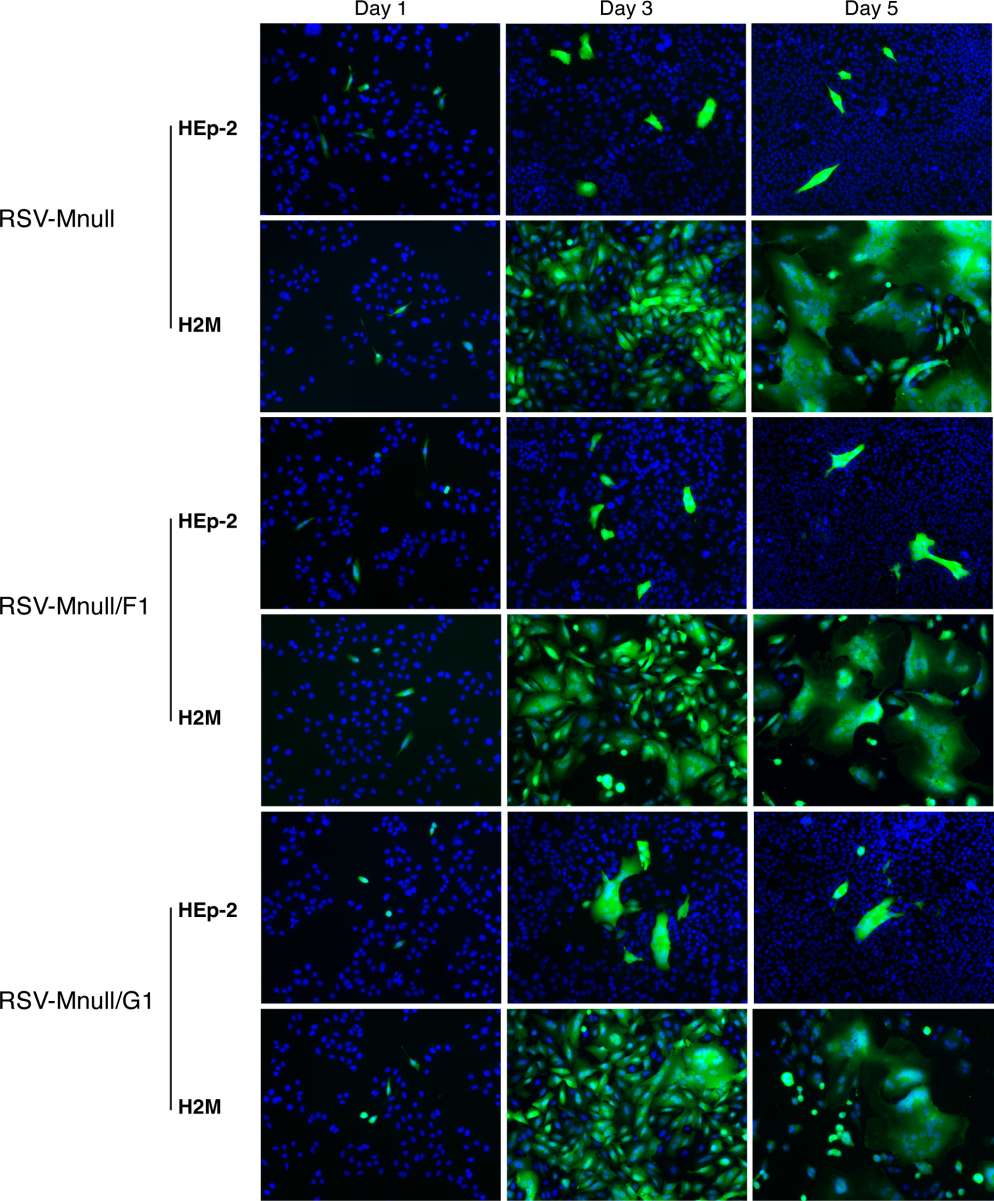
Mnull viruses do not transmit to neighboring cells. HEp-2 cells and H2M (production line) cells were infected with different Mnull viruses at 0.01 PFU/cell, and monitored for 5 days pi. On day 1–5 postinfection, cells were fixed and nuclei were stained using Hoechst stain. To document spreading virus, images were taken on a fluorescent microscope (100x magnification).

We previously demonstrated that the prototype RSV-Mnull was unable to generate infectious progeny *in vivo* after vaccination (25). To verify that the 2nd generation RSV-Mnull viruses were deficient in cell-cell transmission, the M-producing cell line H2-M, as well as parental HEp-2 cells were plated side by side and infected at 0.01 PFU/cell. After incubation at 37 °C, viral spread was monitored with a fluorescent microscope for five days using GFP expression as a marker (**Figure 2**). In H2-M cells, RSV-Mnull viruses spread efficiently, leading to widespread GFP expression and extensive syncytia formation. This is in agreement with typical observations when we generate infectious virus stocks. In contrast, in HEp-2 cells, RSV-Mnull viruses failed to transmit to neighboring cells, resulting in an intact over-confluent monolayer with only a few lingering GFP-positive cells by day 5 pi. No differences were observed between RSV-Mnull, RSV-Mnull/G1 and RSV-Mnull/F1. Thus, as intended, the Mnull viruses are infectious, replicate their genomes, and express genes, but fail to spread to neighboring cells in regular cell types.

### Mnull viruses RSV-Mnull/F1 and RSV-Mnull/G1 express lower levels of NS1

**Figure 3:**
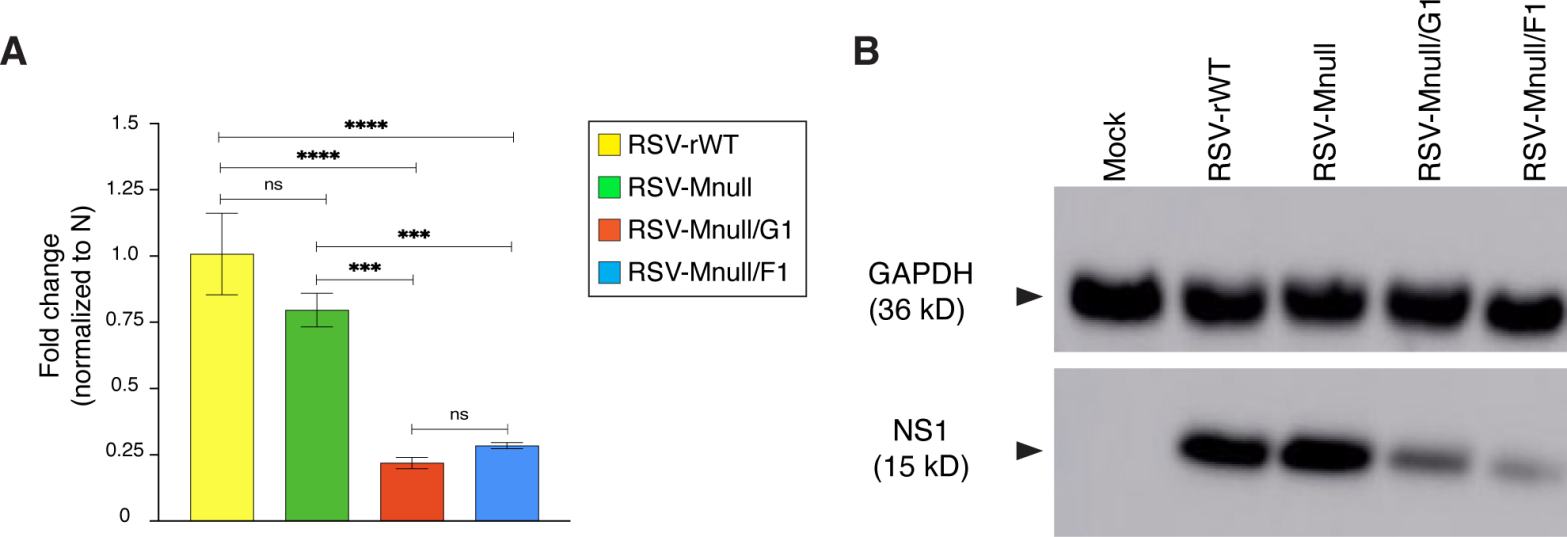
The impact of NS1 relocation on NS1 expression levels. A549 cells were infected with RSV-Mnull/G1, RSV-Mnull/F1, RSV-Mnull, and RSV-rWT, or left uninfected (mock). Infected cell supernatant was examined at 24 hpi. **(A)** Total RNA was extracted and real-time PCR was performed (primer pairs listed in **Table 1**). NS1 levels were normalized to N protein mRNA levels. Fold change is indicated on the Y-axis. ***p<0.001, ****p<0.0001. Error bars are the standard deviation of the mean of triplicate samples. **(B)** Cell lysates were electrophoresed on reducing SDS-PAGE and western blots were generated. Blots were incubated with anti-NS1 Ab and anti-GAPDH Ab (loading control) and developed with ECL.

Next, we examined whether NS1 expression was reduced in cells infected by RSV-Mnull/F1 and RSV-Mnull/G1, by measuring mRNA expression (real-time PCR) and protein expression (western blot). RNA was extracted from infected A549 cells, and mRNA levels were measured at 24 hpi (**Figure 3A**). Both RSV-Mnull/G1 and RSV-Mnull/F1 exhibited significantly reduced NS1 levels compared to the prototype RSV-Mnull and RSV-rWT. Western blot analysis of infected A549 cell lysates was in agreement with the realtime PCR results and showed reduced NS1 protein levels in cells infected with RSV-Mnull/G1 and RSV-Mnull/F1 (**Figure 3B**). Together, the results show that relocation of the NS1 ORF to the eighth genome position significantly reduced NS1 expression.

### Low NS1 levels increase IFN-β but not IFN-λ3

**Figure 4:**
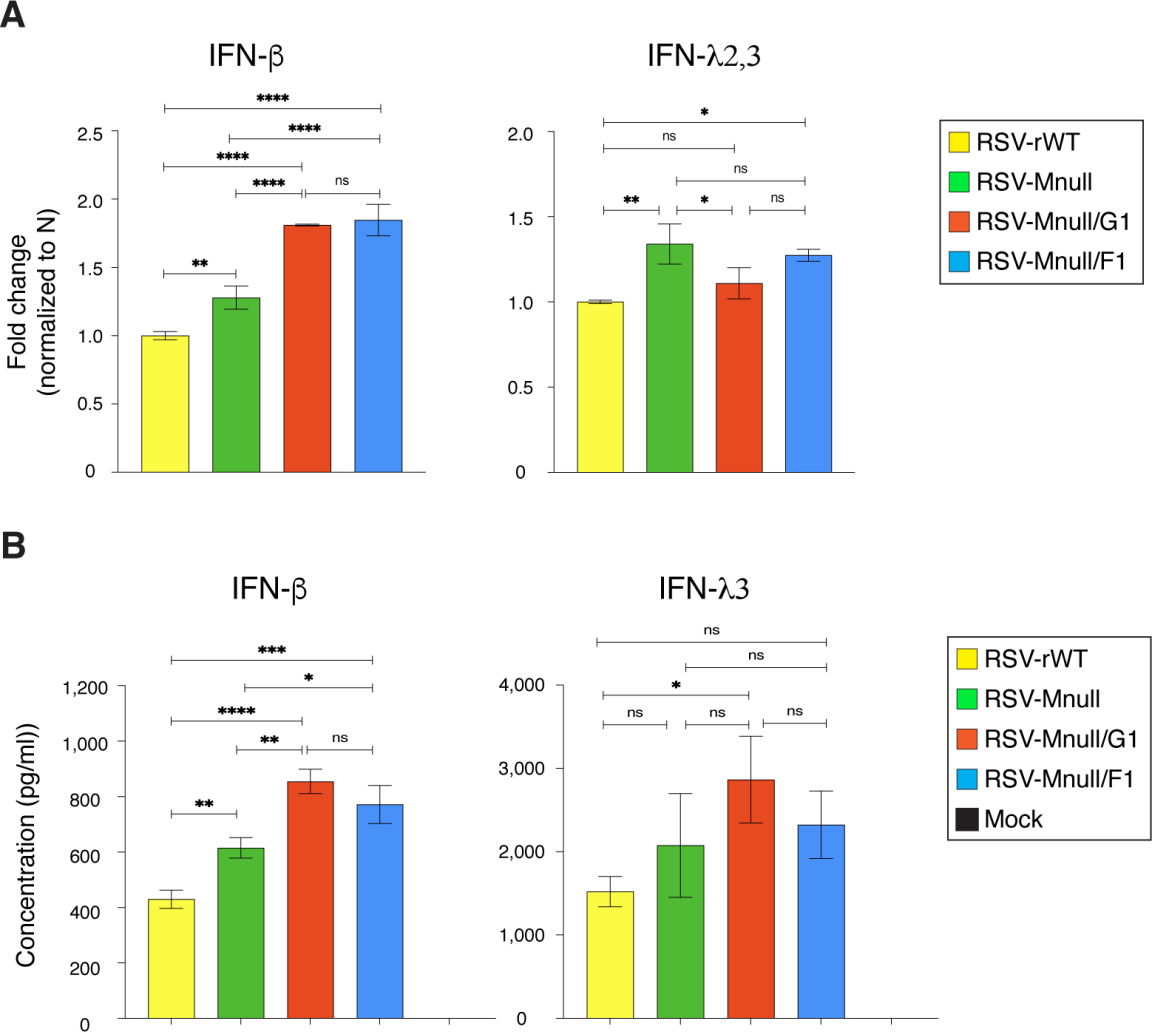
Impact of reduced NS1 levels on IFN expression. A549 cells were infected with RSV-Mnull/G1, RSV-Mnull/F1, RSV-Mnull, and RSV-rWT, or left uninfected (mock). Infected cell supernatant was examined at 24 hpi. **(A)** mRNA levels. Total RNA was extracted from infected cells and real-time PCR was performed (primers listed in Materials and Methods). IFN levels were normalized to RSV N protein mRNA levels. Fold change is indicated on the Y-axis. **(B)** Cytokine ELISA. Supernatants were harvested from infected cells, and cytokine levels were determined using commercially available human IFN-β or IFN-λ3 specific kits. Error bars are the standard deviation of the mean of triplicate samples. *p<0.05, **p<0.01, ***p<0.001, ****p<0.0001.

In the next step, we examined whether reduced NS1 levels had an impact on IFN expression by evaluating the mRNA and protein levels of type I (IFN-β) and type III (IFN-λ2/3) IFNs in infected cells (**Figure 4**). Since IFN-λ2 and IFN-λ3 share 98% sequence identity, a single primer set was designed that targets both λ forms. A549 cells were infected at 1 PFU/cell and analyzed at 24 hpi. mRNA levels were determined by real-time PCR (fold change, normalized to RSV N expression) (**Figure 4A**). Protein levels were determined using commercially available IFN ELISA kits specific for IFN-β or IFN-λ3 (**Figure 4B**). In almost all cases, RSV-Mnull displayed higher IFN levels than RSV-rWT, consistent with recent findings that M acts as an IFN antagonist (27). Relative to RSV-Mnull, RSV-Mnull/G1 and RSV-Mnull/F1 infected cells had significantly increased IFN-β levels, both at the RNA and protein level. However, no differences in IFN-λ2/3 levels were observed among the groups. Together, the data show that IFN-β levels were impacted by lowering NS1 levels.

### Reduced NS1 levels may enhance the anti-viral state

**Figure 5:**
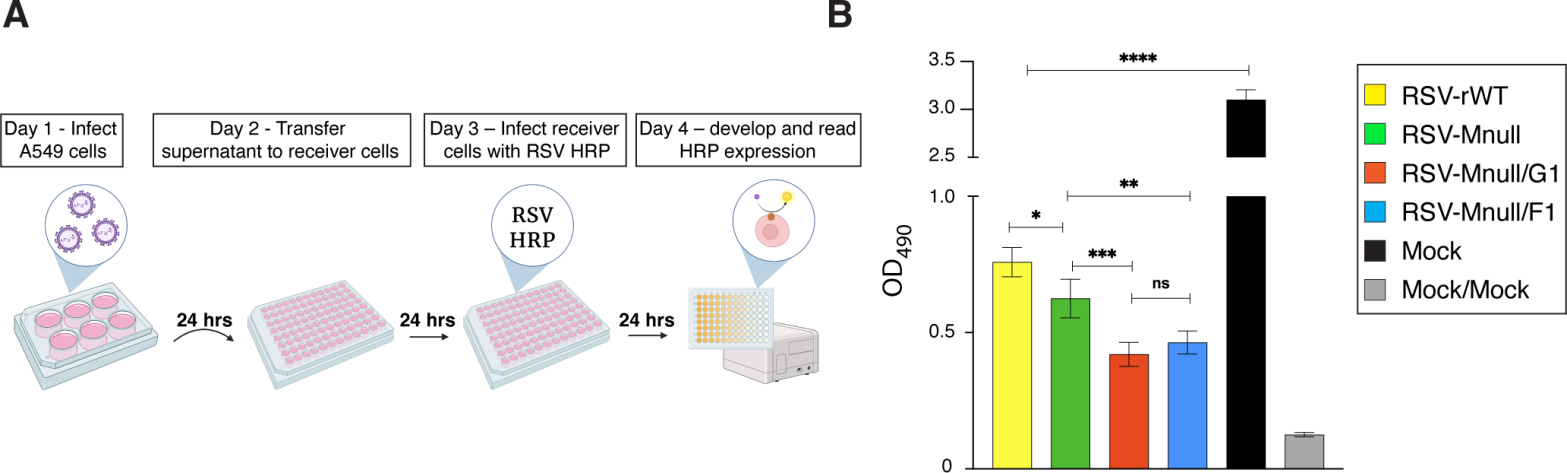
Viruses with enhanced IFN expression and induction of anti-viral state. **(A)** Schematic of anti-viral state assay. An assay was developed to examine whether relocation of NS1 impacts the ability to induce anti-viral state, as an indicator of functional impact. In this assay, supernatants from virus-infected A549 cells are harvested at 24 hpi and incubated for 2 hours on freshly plated receiver A549 cells to induce anti-viral state, followed by infection with a virus carrying an HRP marker gene (RSV-HRP) at 1 PFU/cell. At 24 hpi, the amount of expressed HRP is determined by ELISA as a measure of virus replication. **(B)** Supernatant from A549 cells infected with RSV-Mnull/G1, RSV-Mnull/F1, RSV-Mnull, and RSV-rWT, or left uninfected (mock) were used in the assay described above. Mock-Mock are receiver cells not treated with cell supernatant and left uninfected. Y-axis: OD_490_. Error bars are the standard deviation of the mean of triplicate samples. *p<0.05, **p<0.01 ***p<0.001, ****p<0.0001.

To examine functional impact of altered IFN-β levels, we examined the ability of secreted IFNs to induce anti-viral state in uninfected cells. An assay was developed based on Vlaspolder et al. (1989) and Voigt et al. (2013) (57, 58) (**Figure 5A**). First, A549 cells were infected at 1 PFU/cell with RSV-Mnull/G1, RSV-Mnull/F1, RSV-Mnull, and RSV-rWT, or left uninfected (mock). At 24 hpi, supernatants were clarified by centrifugation at high g-force and incubated for 24 hours on freshly plated receiver A549 cells. Next, cells were infected at 1 PFU/cell with a previously described virus that expresses horseradish peroxidase (HRP) as a marker gene (RSV-HRP) (24). At 24 hpi, OD_490_ levels were determined as previously described (23, 24) as a measure of viral replication. As expected, the negative control (cells treated with supernatants from mock-infected cells) allowed robust infection with RSV-HRP (**Figure 5B**). In contrast, all supernatants of infected cells reduced HRP signals, indicative of a level of anti-viral state. Notably, supernatant from NS1 downregulated Mnull viruses RSV-Mnull/G1 and RSV-Mnull/F1, which induced higher IFN-β levels in **Figure 4**, reduced HRP levels significantly more than RSV-Mnull. Thus, a virus expressing NS1 from the eighth genome position established a stronger antiviral state in receiver cells than the parent virus RSV-Mnull. As G was reported to be an IFN-antagonist (26, 38, 63), having G at first genome position is unlikely to be responsible for the enhanced antiviral state by virus RSV-Mnull-G1, suggesting that NS1 is responsible. In addition, supernatant from RSV-Mnull infected cells reduced HRP levels more than RSV-rWT, consistent with IFN-β levels shown in **Figure 4** and the role of M as an IFN-antagonist. Together, the data reveal that manipulation of NS1 levels in a live single-cycle vaccine can enhance IFN-β levels which in turn may impact antiviral state.

### Induction of anti-viral IgG in serum of vaccinated mice, and neutralization potential

**Figure 6:**
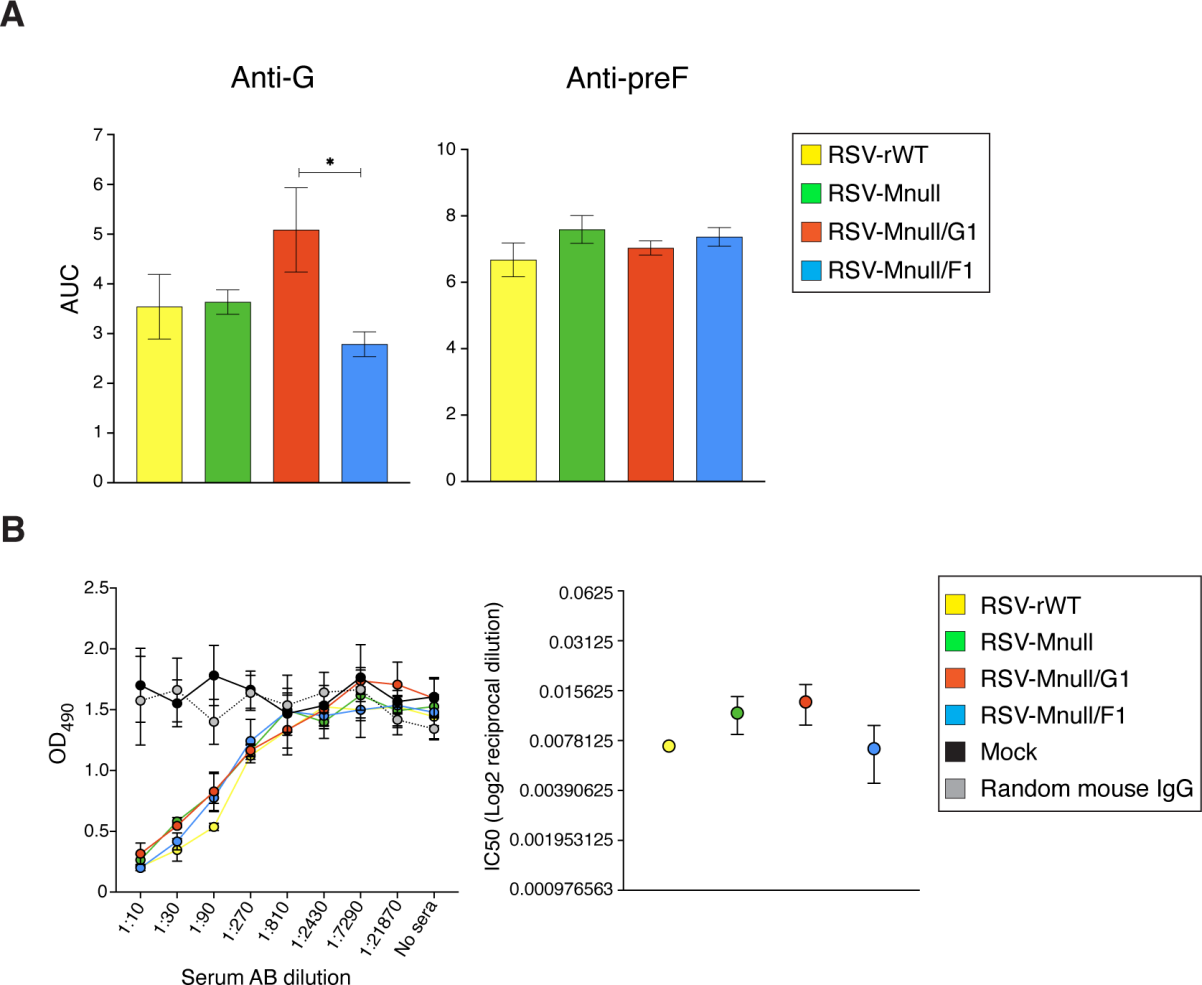
Serum IgG titers and neutralization capacity. **(A)** Eight-week old BALB/c mice were prime-boost vaccinated as described above, and anti-G and anti-preF serum Ab titers were determined three weeks after the boost by ELISA, using purified preF and G protein coated to assay plates. From the curves, Ab levels were quantitated by AUC. Error bars represent the standard error of the mean AUC derived from five individual mice (*p<0.05). **(B)** Mouse sera were heat-inactivated and their neutralization potential was determined using RSV-HRP. Briefly, RSV-HRP was incubated with dilutions of pooled mouse sera (n=5), and then used to infect HEp-2 cells. Random mouse IgG and sera from mock-vaccinated mice were included as negative controls. At 48 hpi, relative HRP levels were determined by ELISA. Curve-fit analyses were performed using Prism10 to determine reciprocal titers that achieved 50% neutralization of RSV-HRP. The data points represent the mean of triplicate samples of pooled sera from 5 mice/group.

In previous work with RSV-Mnull, a single dose of 1 million PFU/mouse induced anti-RSV Abs and protected mice from weight loss after RSV challenge (25). In separate studies with another single-cycle virus, we established that prime-boost vaccination with 0.5 million PFU/mouse was optimal for Ab induction (24). Since our vaccines can potentially be applied to all age groups, initial characterization of the newly designed vaccines was done in adult mice. In **Supplementary Figure S1**, we compared different dose regimens to establish optimal dose for Mnull viruses. We found that a prime-boost regimen of 0.5 million PFU per dose yielded the highest serum Ab concentrations. Using this regimen, eight-week-old BALB/c mice were vaccinated intranasally with RSV-Mnull, RSV-Mnull/G1, RSV-Mnull/F1, RSV-rWT or mock-vaccinated, and boosted 3 weeks later. Three weeks post-boost, blood and perfused lung samples were collected. Anti-G and anti-preF IgG Ab levels in serum were examined using RSV-specific ELISA as previously described (23, 24, 59), and quantified by generating curves based on serial Ab dilutions and calculating the Area Under the Curve (AUC) (**Figure 6**). Overall serum IgG levels were highly similar, with the exception of RSV-Mnull/G1, which induced moderately higher anti-G Ab levels than the other viruses and significantly higher levels of anti-G Abs than RSV-Mnull/F1. This was in agreement with our previous observation that moving the G ORF to the first genome position enhances anti-G Ab levels in mice (23). In contrast, moving F to the first position did not raise anti-preF serum Ab levels and moderately reduced anti-G Ab levels (**Figure 6A**).

We also examined neutralizing potential of the serum Abs, using RSV-HRP, as previously described (24). RSV-HRP was incubated with dilutions of pooled mouse sera (n=5) and then used to infect HEp-2 cells. At 48 hpi, relative HRP expression was determined by ELISA, and IC_50_ values were calculated. The data show that all viruses neutralized equally, consistent with the similar Ab levels observed in **Figure 6A**.

### IgA Abs in the lungs of vaccinated mice

**Figure 7:**
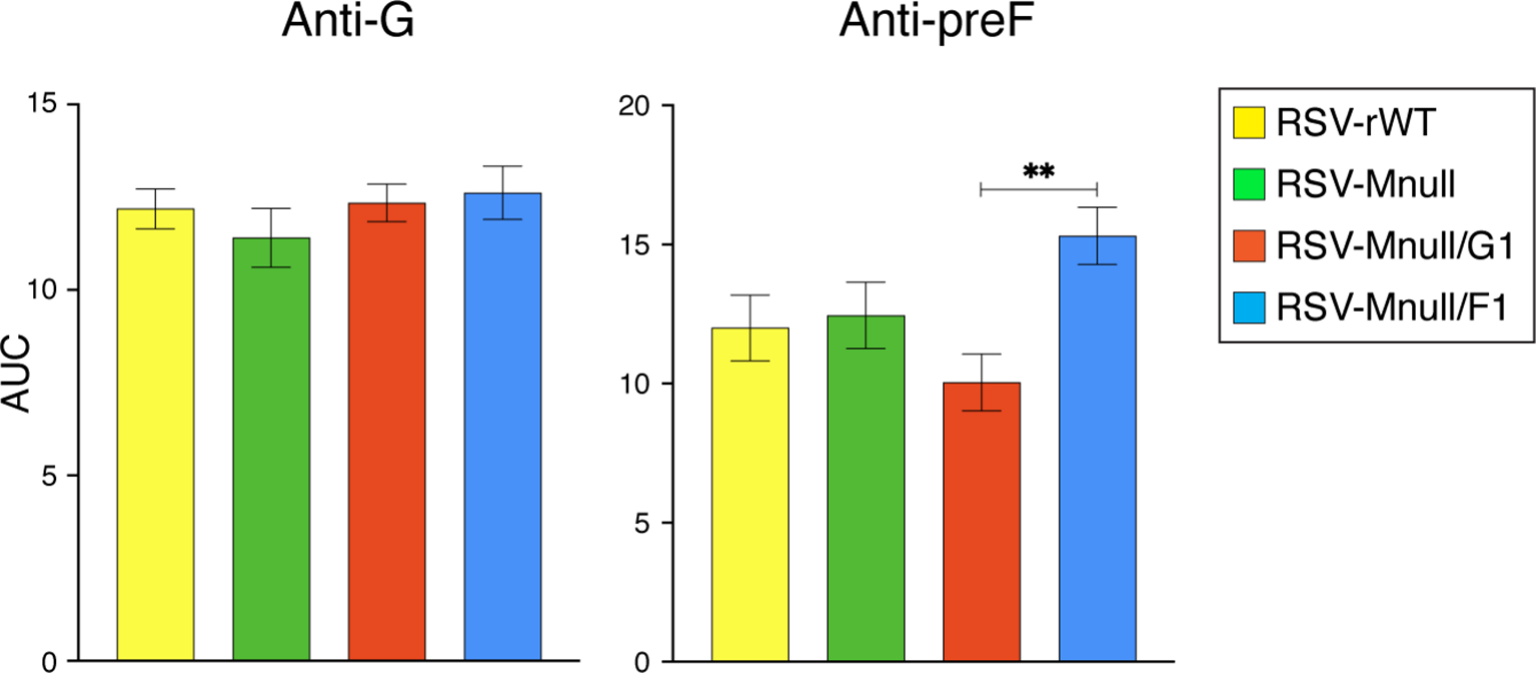
Lung IgA Ab titers at three weeks post-boost. From animals vaccinated in **Figure 6**, lungs were harvested at three weeks post-boost, homogenized, and used to examine anti-preF and anti-G lung IgA levels. Ab levels were determined as described above, with the secondary Ab being an anti-IgA isotype Ab. From the curves, Ab levels were quantitated by AUC. Error bars represent the standard error of the mean AUC derived from five individual mice (**p<0.01).

In addition to serum IgG, we determined lung IgA levels, as some studies suggest a significant association between mucosal RSV-specific immunoglobulins, particularly IgA, and decreased rates of RSV infection and disease severity (11, 64, 65). Perfused lung samples obtained from the immunization study described in the serum IgG Ab analysis were homogenized, centrifuged to remove debris, and the resulting supernatants were used for IgA quantification. Ab levels were determined as above, with the exception that the secondary was an anti-IgA isotype Ab (**Figure 7**). Anti-G IgA levels in the lungs were very similar between RSV-Mnull, RSV-Mnull/G1, RSV-Mnull/F1 and RSV-rWT. Anti-preF IgA levels were also similar between the viruses, with the exception that RSV-Mnull/F1 had moderately higher anti-preF IgA levels, which were significantly different than RSV-Mnull/G1. Thus whereas G at 1st position enhanced serum anti-G IgG levels relative to F at first position (see **Figure 6**), it did not enhance IgA levels in the lung. In contrast, moving F to the first position (RSV-Mnull/F1) did not raise anti-F serum IgG levels but did increase the level of anti-F lung IgA, relative to RSV-Mnull/G1.

### Protection from lung pathology after challenge with wildtype RSV

**Figure 8:**
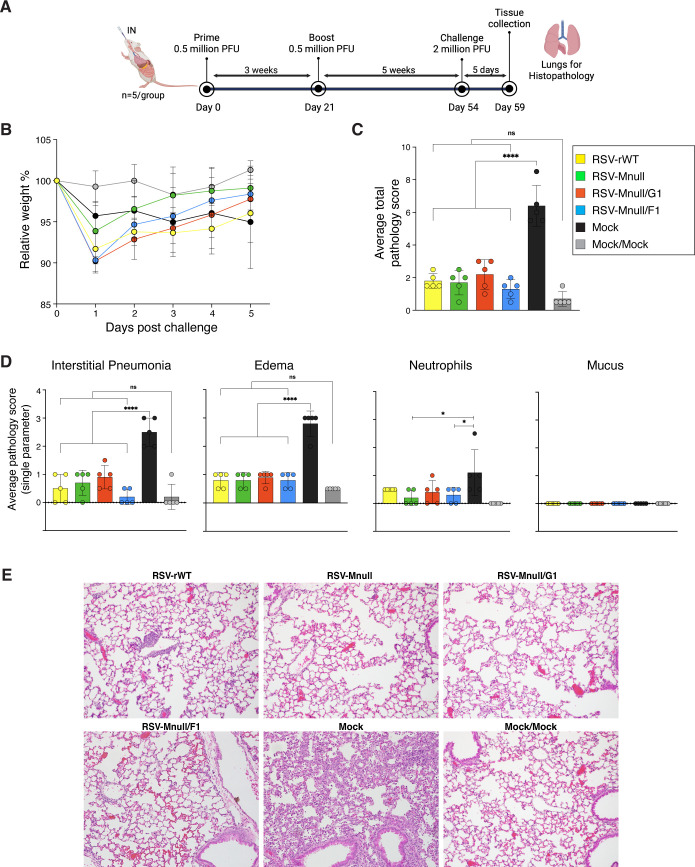
Protection from challenge with wildtype RSV. **(A)** Schematic overview of the experiment. Prime-boost vaccinated mice were challenged with wildtype RSV at 5 weeks post-boost. **(B)** Weight loss post-challenge. Mice were weighed daily. Weights relative to day 0 are shown. **(C, D)** Lung tissues were examined and scored blindly by an ACVP-certified veterinary pathologist for four parameters commonly assessed for RSV-induced pathology (see text), scoring each parameter from 0 (no pathology) to 3 (high pathology). For each group, the average total pathology score is shown in **(C)**; Individual scores are shown in **(D)**. Mock = mock-vaccinated and challenged. Mock/mock = mock-vaccinated and mock-challenged (mock is supernatant from cells harvested identically to virus stocks). Error bars represent the standard deviation of the mean (n=5). *p<0.05, **p<0.01, ***p<0.001, ****p<0.0001, ns, non-significant). **(E)** Images of H&E stained lung sections representative of the scores in panels **(C, D)**.

We previously showed that a single dose of RSV-Mnull induced RSV specific Abs and T cells in mice and reduced viral lung load after challenge (25). However, the ability of RSV-Mnull vaccination to protect from lung pathology, an important indicator of protection, was not examined. In this experiment, mice were prime-boost vaccinated as described above and subsequently challenged with 2 million PFU of RSV-rWT, five weeks after the boost (**Figure 8A**). The negative control group consisted of mice vaccinated with mock material derived from uninfected H2-M cells and processed similarly. Additionally, one group was included that received two mock vaccinations and a mock challenge (Mock/Mock) and thus was without any virus exposure. Mouse weights were monitored daily post-challenge (**Figure 8B**). All mice from vaccinated groups showed some weight loss relative to mice never exposed to RSV (Mock/Mock), and weight loss induced in Mnull virus vaccinated mice was on par with that of RSV-rWT vaccinated mice. Five days post-challenge, lungs were harvested and processed for histological examination using H&E staining. Tissues were examined and scored blindly by an ACVP-certified veterinary pathologist for parameters associated with RSV-induced pathology: interstitial pneumonia (thickness of alveolar septa; leucocytes in the alveolar space), edema (flooding of lymph vessels around blood vessels and airways), mucus and neutrophil influx. As expected, mock-vaccinated mice had the highest average total pathology scores (**Figure 8C**). Vaccination with all other viruses including RSV-rWT equally protected the lungs, reducing average total lung pathology and individual parameters (**Figure 8D**) close to levels seen in the negative control group (Mock/Mock). One potential difference relates to neutrophil scores which were slightly lower for RSV-Mnull and RSV-Mnull/F1 groups relative to mock-vaccinated mice (p<0.05).

### Lung cytokine profile after prime-boost vaccination

**Figure 9:**
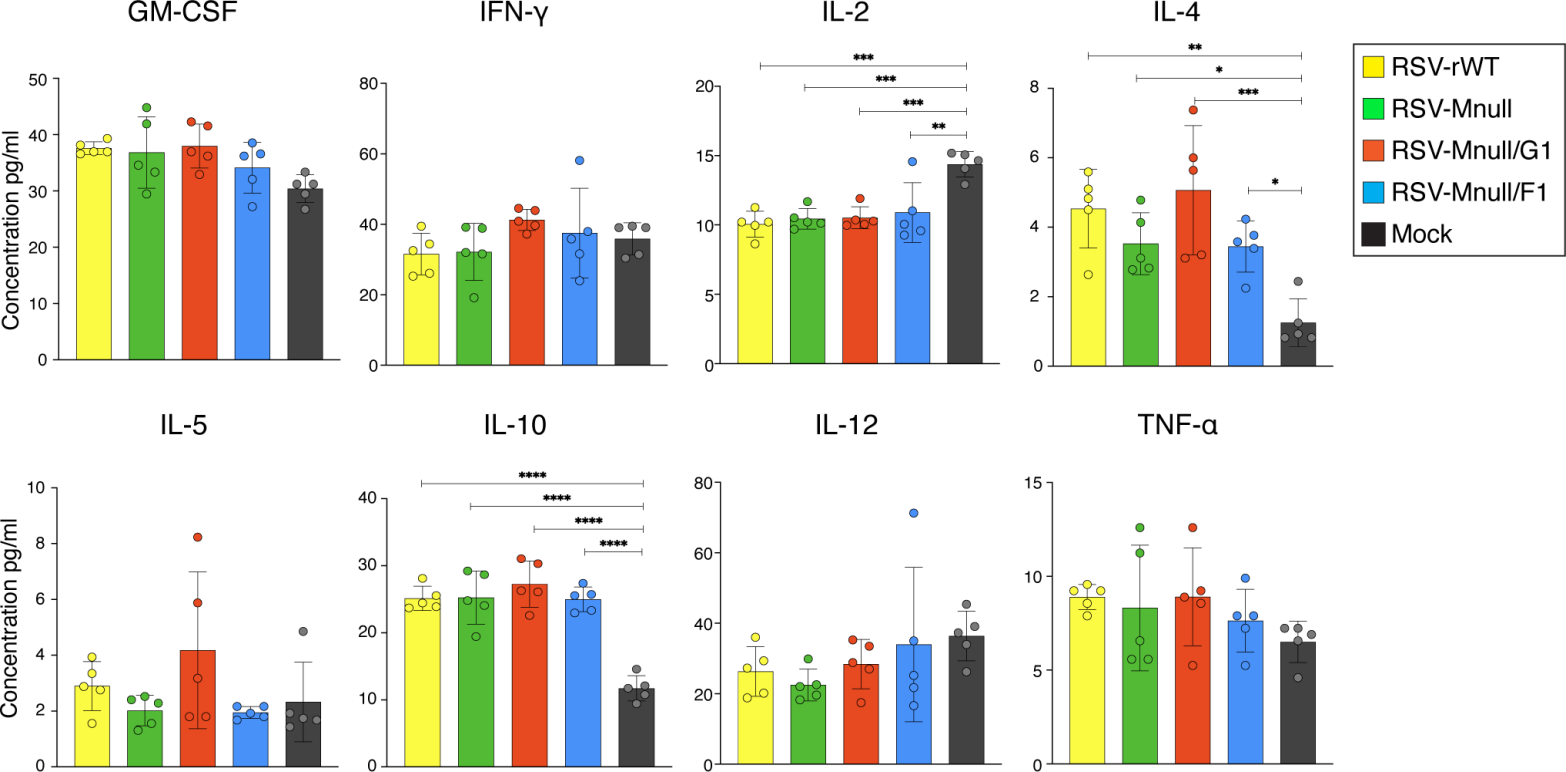
Lung cytokine milieu after prime-boost vaccination. Eight-week old BALB/c mice were prime-boost vaccinated as described above, and lungs were perfused and homogenized four days after the boost. Lung samples were examined with a multiplex immunoassay using a Th1/Th2 cytokine panel with eight cytokines. Cytokine concentrations are indicated on the Y-axis. Mock, mock-vaccinated animals. Error bars represent the standard deviation of the mean (n=5). *p<0.05, **p<0.01, ***p<0.001, ****p<0.0001).

One of the potential advantages of live RSV vaccines is that they have never shown indications of VED in clinical trials of seronegative infants (7, 17, 20, 45). The lung cytokine profile has been used in the RSV field as an indicator of Th1/Th2 (im)balance and VED. Here we examined the cytokine milieu after prime-boost vaccination as a potential indicator of vaccine safety. Mnull viruses were compared to RSV-rWT, which typically induces a cytokine profile that does not result in VED. Animals were prime-boost vaccinated as in **Figure 6**, and lungs were harvested at day 4 pi. Lungs were perfused with PBS, homogenized, and clarified by high-speed centrifugation. Lung samples were analyzed with Bio-Plex using the Pro Mouse Cytokine Th1/Th2 immunoassay kit following the manufacturer’s instructions. The analysis included GM-CSF, IFN-γ, IL-2, IL-4, IL-5, IL-10, IL-12, and TNF-α (**Figure 9**). For most cytokines, no differences were observed between vaccinated and mock groups, suggesting that these responses are largely not specific to RSV. In general agreement with previous work (18), IL-2 levels were significantly lower in all vaccinated groups compared to the mock group, whereas IL-4 and IL-10 levels were elevated. Importantly, none of the measured cytokines differed significantly between the Mnull viruses and RSV-rWT. Multiple previous studies (66–70) have demonstrated that, unlike FI-RSV, wildtype RSV vaccination induces a more balanced cytokine response and does not result in VED after challenge. Together with the lung pathology findings, these results suggest that Mnull viruses, like wildtype RSV, do not predispose to VED after RSV challenge.

### Anti-G and anti-preF IgA in the lungs of vaccinated mice at four days post-boost

**Figure 10:**
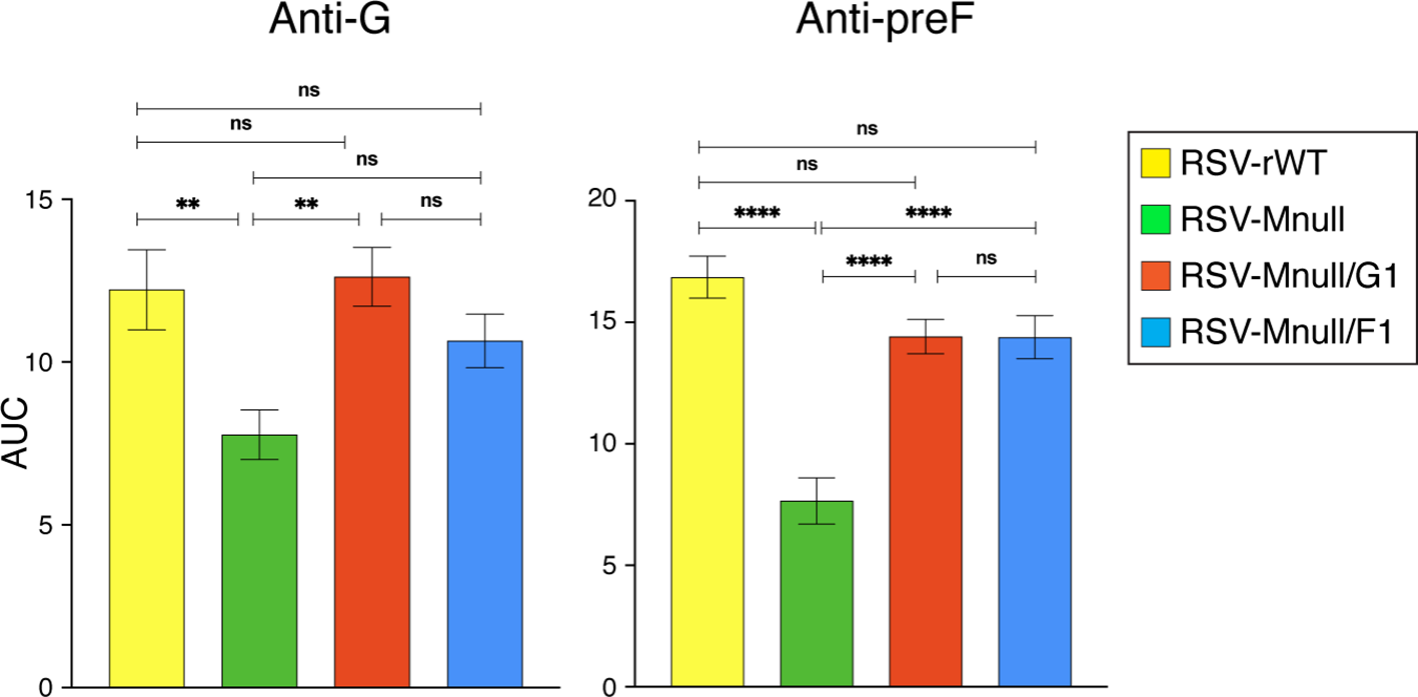
Lung IgA Ab titers at four days post-boost. The levels of anti-preF and anti-G IgA were examined in lungs harvested and homogenized in the cytokine studies of **Figure 9**. Ab levels were determined as described above, with the secondary Ab being an anti-IgA isotype Ab. From the curves, Ab levels were quantitated by AUC. Error bars represent the standard error of the mean AUC derived from five individual mice (**p<0.05, ****p<0.0001, ns, not significant).

PBS perfused lungs harvested 4 days post-boost were analyzed to assess early mucosal IgA responses (**Figure 10**). At this time point, RSV-rWT induced significantly higher lung IgA levels than RSV-Mnull, indicating that intrinsic differences between these viruses affect IgA induction. In contrast, RSV-Mnull/F1 and RSV-Mnull/G1, in which NS1 is relocated to genome position eight, elicited IgA responses comparable to RSV-rWT and significantly greater than RSV-Mnull for both anti-G and anti-preF IgA, reflecting more rapid early IgA induction. These differences were not sustained at 21 days post-boost (**Figure 7**), when lung anti-G and anti-preF IgA levels were largely similar across groups, with only a modest increase in anti-preF IgA observed in the RSV-Mnull/F1 group relative to RSV-Mnull/G1. Importantly, no significant differences were detected between RSV-Mnull and the next-generation vaccine candidates at this later time point, indicating that the distinctions observed at day 4 reflect differences in early IgA induction kinetics.

## Discussion

In this study, we modified a live single-cycle RSV vaccine, which could theoretically be targeted to any age group, in order to enhance IFN induction for the long-term purpose of increasing efficacy. This approach was largely based on previous observations showing that low or delayed type I IFN levels have a negative impact on RSV and other viral diseases, in some cases in a neonatal setting (33, 35, 37–39, 42, 43). For example, Cormier et al. showed that exogenous addition of type I IFN improves RSV disease outcome in neonatal mice (35). In spite of immunologic immaturity, another previous study suggested that young infants are capable of inducing protective levels of neutralizing Abs, after infection with RSV (71). Therefore, a vaccine that enhances early type I or type III IFN and thereby the downstream immune responses, could potentially overcome limitations of immunologic immaturity and be effective in infants. Multiple studies have demonstrated that manipulation of NS1 expression represents an effective strategy to modulate IFN responses and to attenuate RSV. NS1 functions as a major antagonist of type I and type III IFN signaling by disrupting pathways involved in antiviral gene induction (72). Complete deletion of NS1 has been shown to increase IFN signaling and reduce viral replication, although this approach can lead to overattenuation (73) and difficulties propagating vaccine stocks (31, 32). Alternative strategies have therefore focused on partial suppression of NS1 levels. Codon deoptimization of NS1 and NS2 has been shown to reduce NS protein expression, decrease STAT2 degradation, and enhance IFN-mediated antiviral responses, while maintaining strong immunogenicity and genetic stability (74). Similarly, structure-based mutational studies have demonstrated that targeted amino acid substitutions within NS1 can generate graded attenuation phenotypes associated with increased IFN and cytokine expression (75). In the present study, we employed a distinct strategy to downregulate NS1 expression by repositioning the NS1 gene from the first to the eighth genome position, exploiting the transcriptional gradient characteristic of RSV. We did not manipulate NS1 for the purpose of attenuation, since in our case, attenuation is achieved by a block in cell-cell transmission due to lack of M. In this regard, high titer stocks of RSV-Mnull/G1 and RSV-Mnull/F1 were readily generated in M-producing cells. Rather, the goal was to increase IFN levels by combining the absence of the M gene and relocation of NS1. This approach is designed to reduce, but not eliminate, NS1 expression, and enhance IFN responses while preserving sufficient viral replication and antigen expression to support immunogenicity. Here, we found that viruses RSV-Mnull/F1 and RSV-Mnull/G1 had reduced NS1 expression and increased type I IFN expression. In agreement with a recent report on the M protein (27), we also found that the M protein is itself an IFN antagonist. Thus, viruses RSV-Mnull/F1 and RSV-Mnull/G1 carry two modifications that impact IFN induction, and these viruses were characterized and examined for their ability to induce Abs and protect against RSV challenge.

### Mnull viruses with reduced NS1 expression enhance only type I IFN (IFN-β) production

As intended, viruses RSV-Mnull/F1 and RSV-Mnull/G1 had reduced NS1 levels (**Figure 3**), and consequently, enhanced IFN-β levels (**Figure 4**). Supernatants from infected cells also enhanced anti-viral state in uninfected cells (**Figure 5**). However, lower NS1 levels did not enhance type III IFN (**Figure 4**). This was unexpected since NS1 was previously shown to inhibit both type I and type III IFNs in the same cell type used here (A549) (30, 41). In the Spann study (30) however, the biggest impact was when both NS1 and NS2 were deleted. In our case, NS2 remained intact and NS1 was downregulated. It is therefore possible that further downregulation of NS1 and NS2 levels could lead to an increase in IFN-λ as well. At this time, we do not know whether upregulation of IFN-β alone would be sufficient to enhance efficacy in humans.

### Induction of serum IgG Abs

Experimental viruses induced serum Abs at levels similar to, or higher than, RSV-rWT. Moving G to position one enhanced anti-G serum Ab levels, which agreed with our previous study that examined a preF-based single-cycle vaccine (23, 24). In the latter study, placing G at first position did not raise G protein levels at the cell surface of infected cells, but did increase the G protein levels in virions. Exogenous G also enhanced G Ab levels, leading us to speculate that either the input G protein, enhanced G presentation, or improved virus entry (higher infectivity) were responsible for the increases in serum anti-G Abs. However, serum anti-F Abs did not simultaneously increase, suggesting that the serum anti-G Ab levels increased due to higher input quantity of G protein or due to improved presentation of G after *de novo* synthesis from the first genome position. This phenomenon appears specific to G, because moving F to the first genome position did not raise serum anti-F Ab levels. In contrast, RSV-Mnull/F1, but not RSV-Mnull/G1, changed the ratio of IgA Abs in the lungs, with RSV-Mnull/F1 having a higher ratio anti-preF:anti-G IgA Abs than RSV-Mnull/G1 (**Figure 7**). Combined, these findings suggest that anti-G and anti-F Ab induction within one immunological ‘compartment’ (systemic or lung) differ mechanistically, in addition to differences between lung or systemic compartments.

### Protection from challenge

Although not significant, some differences in weight loss were observed, with weight loss after challenge being moderately higher for RSV-Mnull/G1 and RSV-Mnull/F1, relative to RSV-Mnull. Increased IFN levels have previously been linked to greater weight loss during RSV infection (76) and may be responsible for increased weight loss induced by RSV-Mnull/G1 and RSV-Mnull/F1 vaccination.

All viruses protected equally against challenge RSV-induced lung pathology (**Figure 8**). On the one hand this is a positive outcome, as we had not previously examined lung pathology of RSV-Mnull, and since our experimental vaccines only have a single round of replication. On the other hand, we did not see significant improvements in protection by RSV-Mnull/G1 and RSV-Mnull/F1 over the prototype RSV-Mnull. We did find that NS1 and M manipulation indeed raised IFN-β levels and the potential for anti-viral state, and that downregulation of NS1 enhanced IgA induction and anti-G serum IgG relative to RSV-Mnull. However, as far as protection against pathology, even the RSV-Mnull prototype protected from lung pathology, and our model may not be sufficient to reveal minor differences/improvements by the modifications made. In addition, due to species specificities of IFNs (41, 77), it is possible that RSV-Mnull/G1 and RSV-Mnull/F1 have more impact on immune parameters and protection in humans.

### Lung cytokines

The only observed differences in the lung cytokine profile, were reduced IL-2 and increased IL-4 and IL-10 (**Figure 9**). This was true for all viruses tested including RSV-rWT. Although the ratio of IL-2 to IL-4 was low relative to mock controls, a similar profile was observed following RSV-rWT infection, which is known to induce a balanced immune response without VED (78). FI-RSV–type VED in mice is characterized by exaggerated lung histopathology and worsened clinical disease after challenge. In our study, Mnull-vaccinated animals did not exhibit increased lung pathology or other signs of enhanced disease after RSV challenge compared with RSV-rWT controls. Thus, it appears the Mnull viruses are safe or at least do not appear to induce VED.

### Lung IgA induction

Our long-term goal is to apply our vaccines intranasally, largely due to the potential for mucosal immunity. Therefore, we also examined induction of lung IgA Abs. Importantly, IgA was found to correlate better than IgG with decreased rates of RSV infection and disease severity (11, 64, 65). First, we showed that the single-cycle viruses induced high and very similar levels of anti-G and anti-F lung IgA three weeks after the boost, with the exception that RSV-Mnull/F1 had significantly higher anti-preF IgA levels than RSV-Mnull/G1 (**Figure 7**). Repositioning G to the first genomic position enhanced serum anti-G IgG responses without increasing lung IgA. In contrast, relocation of F to the first position did not augment serum anti-F IgG but was associated with increased lung anti-F IgA. These divergent systemic and mucosal Ab responses likely reflect differences in immune induction pathways between the systemic and mucosal compartments, as well as antigen-specific variation in processing and presentation within these environments.

When lung IgA was examined at an earlier timepoint (4 dpi) (**Figure 10**), we found that RSV-rWT induced significantly higher levels of IgA than RSV-Mnull, suggesting that limited virus transmission/replication in the lung may be required for optimal early IgA induction. Although this is an undesirable outcome for a single-cycle vaccine candidate, relocation of NS1 to genome position eight restored early IgA levels to those observed with RSV-rWT. This indicates that reduced NS1 expression, and presumably increased type I IFN responses, enhances the rate of early IgA induction. By contrast, at the later time point of 3 weeks post-infection, IgA levels were comparable across groups, likely reflecting cumulative IgA production over time that obscured the early differences observed at 4 dpi. Thus, downregulation of NS1 primarily influenced the kinetics of IgA induction rather than the overall magnitude of the IgA response.

### Impacts of gene relocation

In previous work, we found that higher G input levels (both exogenously and by incorporating more G into the virion via genome relocation) enhanced anti-G antibody levels (23). This suggests that antigen level itself contributes to the amount of antibodies induced by a live virus. Whereas RSV transcription does follow a gradient (61, 79), Piedra et al, 2020 showed that neither the viral mRNA nor protein levels strictly match genome location (60). In particular, they found that G is more highly expressed from its native location than expected. Thus, it is possible that the expression levels of G from 1st and native (7th) genome location are more similar than expected, and this may partially explain the observed minimal differences. On the other hand, the differences observed among the three Mnull vaccines in serum IgG and mucosal IgA responses, described above, raise the possibility that genome repositioning influences immunogenicity through mechanisms beyond antigen level. For example, moving a gene closer to the 3’ promoter may alter the spatio-temporal expression of the relocated gene, even if steady state expression levels at a given time postinfection are unchanged. This could include impact on mRNA or protein stability, post-translational modifications, translation efficacy or an altered host response. In the case of G, early high levels could have an impact on the IFN response since G is itself an IFN antagonist (26, 29). In turn, early negative impact on IFN levels may affect the downstream host response. NS1 relocation may also affect virus replication as it appeared to have pro-viral functions independent of its effect on IFN (31, 32), and as such have an indirect impact on the amount of antigen. In short, genome relocation can have diverse impacts, ranging from antigen levels, virion incorporation levels, altered virus replication levels, and IFN levels, all of which can influence antibody induction. Furthermore, the systemic and mucosal compartments process antigens differently, which may explain for example why the anti-G and anti-F IgA have distinct longevities. A better understanding of the mechanisms underlying antigen-specific IgG and IgA may help explain how seemingly equivalent antigen expression *in vitro* could result in distinct systemic IgG and early mucosal IgA responses *in vivo*, and importantly, will help design and tailor vaccines to achieve the best possible antibody level and combination for protection against lung disease.

## In conclusion and future direction

Relative to other live-attenuated approaches, live single-cycle vaccines have a unique safety component as they are blocked in cell-cell transmission and do not generate progeny, and therefore cannot revert to a more virulent virus especially in age-related or other immune-compromised situations. Single-cycle vaccines are however not attenuated for genomic replication or transcription and therefore generate high levels of viral antigens, and are readily produced in complementing production cell lines (24, 25). For our prototypic Mnull vaccine, RSV-Mnull, Ab specificities and protection against lung pathology (which represents a more stringent protection parameter) had not been previously examined. Here, further characterization of RSV-Mnull expanded our previous work in showing G and F-specific IgG and IgA, and lung protection of mice after wildtype RSV challenge, without evidence for VED.

As intended, modified viruses RSV-Mnull/G1 and RSV-Mnull/F1 showed decreased NS1 levels and increased IFN-β induction but did not increase IFN-λ levels. RSV-Mnull/G1 and RSV-Mnull/F1 also enhanced the anti-viral state, showing potential functional impact of NS1 relocation. Placing G or F at the first genome position had some impact on serum IgG and lung IgA levels respectively, and on the ratio of anti-preF:anti-G Abs. Whereas RSV-Mnull unexpectedly reduced IgA levels, relocation of NS1 restored IgA levels to levels observed with wildtype RSV; The latter is significant as IgA can make an important contribution to the prevention of RSV disease (18, 48–51). Such differences, and any roles of genome position are interesting and may help design the Ab component of future RSV vaccines. The underlying mechanisms are however not yet understood and require more study. All viruses neutralized RSV *in vitro* equally and protected animals equally against a high challenge dose of RSV-rWT. However, we could not document major improvements of RSV-Mnull/G1 and RSV-Mnull/F1 over the RSV-Mnull prototype in protection (neutralizing Abs and lung pathology) against RSV. This may be due to limitations of the model, as even the RSV-Mnull itself almost fully protects mice against challenge, and because *in vitro* neutralization does not always fully appreciate the contribution of anti-G Abs due to differences of virus attachment *in vivo* and *in vitro* (80–82).

Nevertheless, we successfully generated RSV-Mnull/G1 and RSV-Mnull/F1, live vaccines with enhanced type I IFN induction and with a single-cycle phenotype that provides a level of safety, does not require tuning, and is thus relatively predictable. Both vaccines were protective and induced higher early IFN levels and higher anti-viral state, which may be of further potential benefit in the natural host. Moreover, relocating G to first position moderately enhanced serum anti-G Ab levels. Although single-cycle replication lowered lung IgA levels, G relocation restored those levels to those induced by RSV-rWT. Based on these findings, we anticipate further developing RSV-Mnull/G1 as a platform for safe and efficacious live vaccines. An important question that remains is whether single-cycle vaccines can also achieve sufficient efficacy in humans. We believe that in the long term, this may be a matter of reducing the impact of virulence factors, and other genetic modifications that enhance immune memory, overcome immune immaturity, and avoid VED especially in the RSV-naive target population.

## Data Availability

The raw data supporting the conclusions of this article will be made available by the authors, without undue reservation.
