# Supplementary material for: Next-generation single-cycle respiratory syncytial virus vaccines with increased type I interferon induction yield robust systemic and mucosal responses in mice
Source: Front Immunol. 2026 Apr 10;17:1778423. doi: 10.3389/fimmu.2026.1778423 (PMC13106312; doi:10.3389/fimmu.2026.1778423)

**Fig S1. Establishment of Optimal Dose Regimen for Mnull virus**

1. Eight-week-old female BALB/c mice were anesthetized with isoflurane and immunized intranasally with RSV-Mnull. Four experimental groups were included. Two groups received a single prime dose of either 0.5 million PFU or 1 million PFU, and serum samples were collected six weeks post-prime. The remaining two groups received a prime–boost regimen with a three-week interval. One group was immunized with 0.5 million PFU for both prime and boost, while the other received a 1 million PFU prime followed by a 0.5 million PFU boost. Samples were harvested three weeks after the boost.
2. The serum IgG antibody concentrations were measured against PreF and G antigens. Prime-only immunization induced relatively low antibody levels, whereas prime–boost regimens showed substantial enhancement. Given that the 0.5 million PFU prime followed by a 0.5 million PFU boost yielded equal or higher antibody levels, it was selected as the optimal dosing approach. Error bars indicate SEM from three mice.


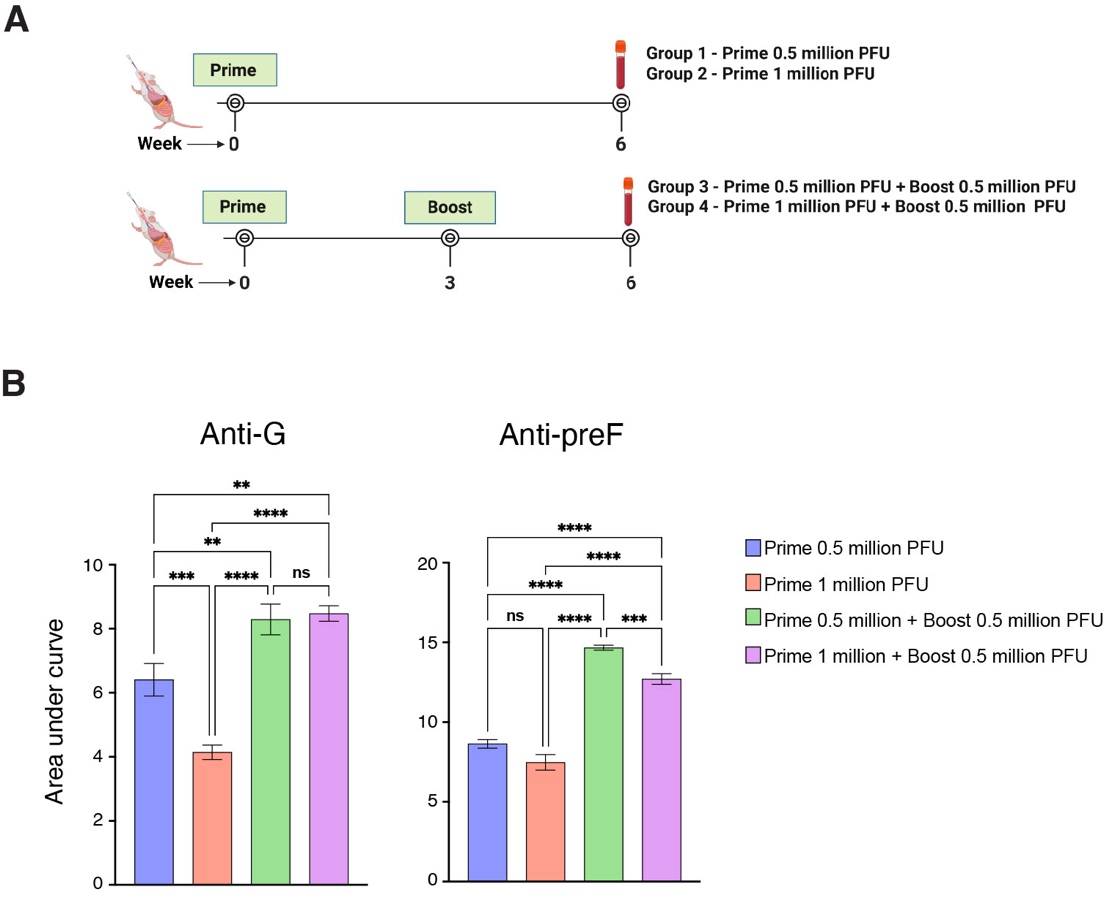

Supplement: Supplementary file 1 [file Table1.docx]
